# Advanced and More Symptomatic B-Cell Non-Hodgkin Lymphoma at Diagnosis After COVID-19 Disruptions in a Romanian Tertiary Center

**DOI:** 10.7759/cureus.109376

**Published:** 2026-05-21

**Authors:** Paul C Toboltoc, Adela Vekony, Gagiu Ioana

**Affiliations:** 1 Pathology, Sibiu County Emergency Hospital, Sibiu, ROU

**Keywords:** b-cell non-hodgkin lymphoma, covid-19 pandemic, delayed diagnosis, hematologic malignancies (hm), stage migration

## Abstract

Background

B-cell non-Hodgkin lymphoma (B-NHL) requires timely tissue diagnosis, staging, and therapeutic planning, the clinical trajectory being shaped from the outset by stage at presentation, tumor burden, symptom burden, and organ involvement; against that background, pandemic-related disruption of non-COVID services raised concern that patients with hematologic malignancies might re-enter care later and in less favorable clinical condition. This study evaluated whether the post-pandemic recovery in case numbers at a Romanian tertiary center was accompanied by a shift toward more advanced and more symptomatic disease at diagnosis.

Methods

This retrospective single-center study included adults newly diagnosed with B-NHL at the Sibiu County Emergency Clinical Hospital between March 11, 2018, and March 7, 2024. The cohort was stratified into pre-pandemic, pandemic, and post-restriction intervals to capture the clinical consequences of successive healthcare contexts. Patients were eligible if they had at least two standard inpatient admissions within the corresponding period, day-hospital admissions being excluded, and records that were incomplete, inconsistent, or clearly erroneous also being removed. Demographic, clinicopathologic, treatment-related, and selected laboratory variables were analyzed.

Results

Of 585 lymphoma records initially identified, 366 met the general eligibility criteria, and 123 met the study-specific criteria for newly diagnosed B-NHL, including 43 pre-pandemic cases, 35 pandemic cases, and 45 post-restriction cases. Although the histopathologic spectrum remained broadly stable, diffuse large B-cell lymphoma continuing to predominate throughout, the post-restriction interval was marked by a redistribution toward more advanced disease, with advanced stage III-IV cases increasing from 25 patients before the pandemic and 21 during the pandemic to 34 after restrictions, while stage IV disease increased from 16 and 13 cases in the first two intervals to 22 cases in the post-restriction interval. Additionally, the proportion of patients without B-symptoms fell from 53.5% and 62.9% in the first two intervals to 11.1% thereafter.

Conclusions

In this cohort, the numerical recovery in newly diagnosed B-NHL cases after the pandemic was accompanied not by full clinical normalization, but by a more advanced and more symptomatic pattern of presentation, the overall findings being compatible with delayed access to timely lymphoma evaluation and with the persistence of downstream effects even after formal service reopening.

## Introduction

B-cell non-Hodgkin lymphoma (B-NHL) comprises a biologically and clinically heterogeneous group of mature B-cell neoplasms, ranging from indolent entities to highly aggressive disease and requiring, from the moment of first presentation, a diagnostic approach that is both morphologically rigorous and clinically well coordinated, because stage at presentation, tumor burden, symptom burden, performance status, and organ involvement all contribute to shaping not only the diagnostic workup itself but also treatment intensity, short-term stability, and eventual prognosis; accordingly, tissue diagnosis, immunophenotyping, staging, and baseline laboratory assessment remain central to early clinical decision-making in B-NHL [1,2].

The COVID-19 pandemic disrupted that pathway in a manner that was at once administrative, organizational, and clinical, with the World Health Organization (WHO) characterizing COVID-19 as a pandemic on March 11, 2020, while Romania had already reported its first laboratory-confirmed case on February 26, 2020. As in many other countries, the Romanian response involved rapid public health restrictions, reorganization of hospital circuits, redistribution of personnel and beds, and a practical de-prioritization of segments of routine non-COVID care during critical periods, thereby altering the conditions under which patients with suspected malignancy could enter specialist evaluation [3-5].

Across oncology, these disruptions were followed by fewer new diagnoses, reduced diagnostic activity, incomplete recovery in some settings, and, in several cohorts, a relative loss of earlier-stage presentations, modeling studies, population-level registry analyses, and systematic reviews consistently raising concern that diagnostic interruption during the pandemic might subsequently translate into more advanced disease at presentation and into less favorable outcomes. Lymphoma-specific data point in the same direction, with the Swedish Lymphoma Register documenting fewer diagnoses during the early pandemic months, with a larger proportion of stage IV disease in 2021, while a Turkish Hodgkin lymphoma cohort reported a significantly longer symptom-to-diagnosis interval during the pandemic [6-14].

Against this background, the present study compared newly diagnosed B-NHL across pre-pandemic, pandemic, and post-restriction intervals in a Romanian tertiary center, seeking not merely to quantify fluctuations in case volume but to determine whether the post-pandemic numerical recovery was accompanied by a shift in stage at diagnosis, B-symptom burden, comorbidity profile, laboratory characteristics, and treatment patterns, thereby clarifying whether recovery in access to care was also reflected in recovery of clinical presentation.

## Materials and methods

Study design and setting

This retrospective observational single-center study included adult patients newly diagnosed with B-NHL at the Sibiu County Emergency Clinical Hospital, Romania, between March 11, 2018, and March 7, 2024. The study population was stratified into three predefined temporal intervals corresponding to the pre-pandemic period, the pandemic period, and the post-restriction period, so that changes in diagnostic capture and clinical presentation could be examined within a stable institutional environment.

**Figure 1:**
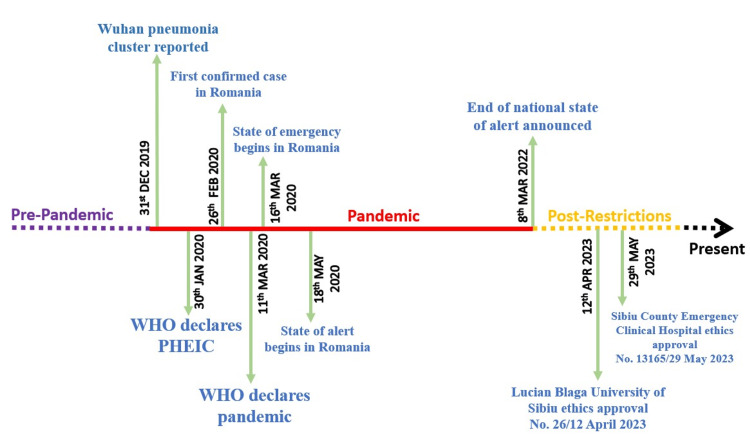
Timeline of the study intervals and key pandemic-related and administrative milestones The timeline displays the three predefined study intervals: pre-pandemic, March 11, 2018, to March 10, 2020; pandemic, March 11, 2020, to March 7, 2022; and post-restriction, March 8, 2022, to March 7, 2024. The figure also marks the first confirmed COVID-19 case in Romania on February 26, 2020, the WHO pandemic declaration on March 11, 2020, and the two ethics approvals granted on April 12, 2023, and May 29, 2023. PHEIC: Public Health Emergency of International Concern, WHO: World Health Organization Created using Microsoft PowerPoint 2016 for Windows (Microsoft Corp., Redmond, WA)

The three temporal intervals were selected a priori to provide two-year comparison windows around the main pandemic disruption. The pre-pandemic period extended from March 11, 2018, to March 10, 2020; the pandemic period extended from March 11, 2020, to March 7, 2022; and the post-restriction period extended from March 8, 2022, to March 7, 2024. The temporal structure of the study, together with the first confirmed COVID-19 case in Romania, the World Health Organization (WHO) pandemic declaration, and the two institutional ethics approvals, is summarized in Figure 1.

Cohort assembly and eligibility criteria

A retrospective census-type sampling strategy was used, and all potentially eligible lymphoma records identified within the predefined study interval were screened for inclusion. The final sample size was therefore determined by the number of records meeting the predefined eligibility criteria.

A total of 585 lymphoma records were initially identified, of which 366 met the general eligibility criteria for lymphoma and underwent further screening; the final cohort consisted of 123 newly diagnosed B-NHL cases, including 43 in the pre-pandemic interval, 35 in the pandemic interval, and 45 in the post-restriction interval. Eligibility required adult age and at least two standard inpatient admissions within the corresponding study period; this threshold was used to ensure a minimally adequate clinical and biological record over time, while day-hospital admissions were excluded because they usually reflect isolated therapeutic or follow-up encounters rather than full inpatient reassessment. Records that were incomplete, internally inconsistent, or clearly erroneous were also excluded.

Inclusion criteria were adult age (over the age of 18), newly diagnosed B-cell non-Hodgkin lymphoma, diagnosis established within one of the three predefined study intervals, availability of a pathology-based diagnosis, and at least two standard inpatient admissions recorded within the corresponding interval. Exclusion criteria were Hodgkin lymphoma, T- or NK-cell lymphoma, prevalent or relapsed cases diagnosed before the relevant interval, day-hospital-only encounters, insufficient documentation to confirm diagnosis or temporal allocation, duplicate records that could not be reconciled, and records with major internal inconsistencies.

Internally inconsistent records were defined as records with discordant patient identifiers across admissions, conflicting diagnosis dates preventing reliable temporal allocation, discordance between the pathology report and the clinical discharge diagnosis, duplicate admissions that could not be assigned to a single patient trajectory, or missing key documentation preventing confirmation of newly diagnosed B-NHL status. Cases with incomplete stage documentation but otherwise confirmed newly diagnosed B-NHL were retained and classified as having unspecified stage, in order to avoid excluding clinically valid cases solely because of incomplete staging notation.

Variables and definitions

Data were retrospectively extracted from admission notes, discharge summaries, pathology reports, and routine laboratory records. The analyzed variables included age, sex, area of residence, occupational status, educational level, marital status, histopathologic subtype, Ann Arbor stage at diagnosis recorded according to the Lugano recommendations for initial evaluation and staging of Hodgkin and non-Hodgkin lymphoma [2], B-symptoms at presentation, associated comorbidities, treatment regimen, and selected laboratory parameters. B-symptoms were defined as fever above 38°C, drenching night sweats, and unintentional weight loss exceeding 10% of body weight within the preceding six months, while comorbidities were grouped by major organ system in order to facilitate structured comparison across the three periods.

Laboratory assessment

The laboratory profile was evaluated at two predefined time points, namely, the first hospitalization corresponding to diagnosis and the last available hospitalization within the study interval. The assessed parameters included hemoglobin, hematocrit, red blood cell count, white blood cell count, platelet count, lactate dehydrogenase, total bilirubin, creatinine, glucose, total cholesterol, triglycerides, and total serum proteins; whenever appropriate, these values were additionally categorized as below normal, within normal limits, or above normal according to local laboratory reference ranges. The institutional reference intervals were as follows: hemoglobin, 13.5-17.5 g/dL in men and 12.0-15.5 g/dL in women; hematocrit, 38.8%-50.0% in men and 34.9%-44.5% in women; white blood cell count, 4-11 × 10³/μL; glucose, 70-99 mg/dL; total bilirubin, 0.3-1.9 mg/dL; creatinine, 0.7-1.3 mg/dL; total cholesterol, <200 mg/dL; and total serum proteins, 6.0-8.3 g/dL. For laboratory parameters for which no sex-specific or numeric interval was available in the extracted institutional reference table, interpretation followed the corresponding range displayed in the original laboratory report.

Statistical analysis

Data were manually extracted from admission notes, discharge summaries, pathology reports, and routine laboratory records, and were entered into a dedicated database created in Microsoft Excel 2016 for Windows. Statistical analyses were performed using GraphPad Prism version 8.0.1 for Windows (GraphPad Software, San Diego, CA) and IBM SPSS Statistics for Windows version 26.0 released in 2019 (IBM Corp., Armonk, NY). Continuous variables were summarized as means and standard deviations (SDs), with additional descriptive indicators used where appropriate, whereas categorical variables were expressed as absolute numbers and percentages. Normality was assessed before selecting parametric or non-parametric tests. Depending on data type, distribution, and pairing structure, comparisons were performed using the chi-square test or Fisher’s exact test for categorical variables, one-way analysis of variance (ANOVA) or Kruskal-Wallis test for continuous variables compared across the three temporal groups, Mann-Whitney test for two-group non-parametric comparisons, and paired t-test or Wilcoxon signed-rank test for paired comparisons between diagnosis and the last available evaluation. Statistical significance was set at an alpha threshold of 0.05.

Ethics

The study was conducted in accordance with the Declaration of Helsinki and applicable data protection regulations. All data were anonymized before analysis, and the retrospective design did not influence diagnostic or therapeutic management. Ethical approval was granted by the Ethics Committee of Lucian Blaga University of Sibiu (number: 26/12 April 2023) and by the Ethics Committee of the Sibiu County Emergency Clinical Hospital (number: 13165/29 May 2023), with waiver of informed consent because of the retrospective and non-interventional nature of the study.

## Results

Cohort selection and histopathologic distribution

**Figure 2:**
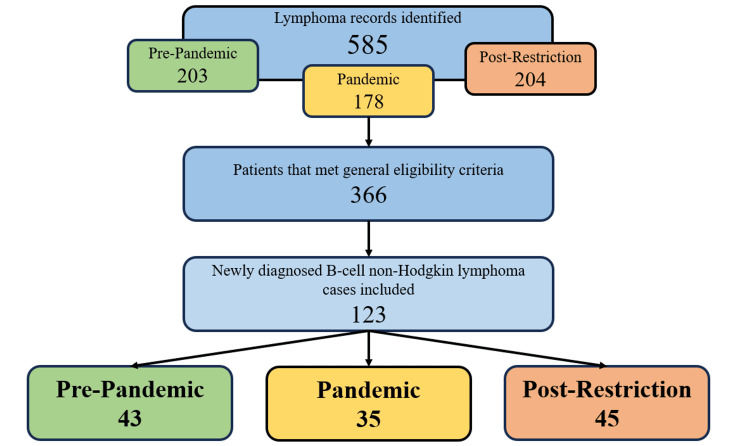
Flow diagram of cohort selection across the three study periods Flow diagram of cohort selection across the three study periods. The figure summarizes the initially identified lymphoma records, the patients meeting general eligibility criteria, and the final newly diagnosed B-cell non-Hodgkin lymphoma cohort stratified by temporal interval. Created using Microsoft PowerPoint 2016 for Windows

During the overall study interval, 585 lymphoma records were identified, including 203 in the pre-pandemic period, 178 in the pandemic period, and 204 in the post-restriction period. After application of the general and study-specific eligibility criteria, 123 newly diagnosed B-NHL cases remained for final analysis, namely, 43 in the pre-pandemic interval, 35 in the pandemic interval, and 45 in the post-restriction interval (Figure 2). The histopathologic spectrum remained broadly comparable across the three periods, with diffuse large B-cell lymphoma being the most frequent subtype throughout and no major qualitative redistribution of entities becoming apparent (Table 1).

**Table 1:** Distribution of histopathologic subtypes of newly diagnosed B-cell non-Hodgkin lymphoma across the three study periods Data are presented as numbers (%) within each study period and overall. Percentages in the Overall column were calculated using the full cohort (N = 123) as the denominator.

Histopathologic subtype	Pre-pandemic (n = 43)	Pandemic (n = 35)	Post-restriction (n = 45)	Overall (N = 123)
Diffuse large B-cell lymphoma	20 (46.5%)	15 (42.9%)	17 (37.8%)	52 (42.3%)
Small lymphocytic lymphoma	9 (20.9%)	9 (25.7%)	15 (33.3%)	33 (26.8%)
Follicular lymphoma	2 (4.7%)	3 (8.6%)	4 (8.9%)	9 (7.3%)
Mantle cell lymphoma	0 (0.0%)	2 (5.7%)	2 (4.4%)	4 (3.3%)
Marginal zone lymphoma	6 (14.0%)	3 (8.6%)	4 (8.9%)	13 (10.6%)
Plasmablastic lymphoma	1 (2.3%)	0 (0.0%)	1 (2.2%)	2 (1.6%)
Lymphoplasmacytic lymphoma	3 (7.0%)	0 (0.0%)	1 (2.2%)	4 (3.3%)
Hairy cell leukemia	2 (4.7%)	3 (8.6%)	0 (0.0%)	5 (4.1%)
Burkitt lymphoma	0 (0.0%)	0 (0.0%)	1 (2.2%)	1 (0.8%)

Stage at diagnosis and B-symptoms

**Figure 3:**
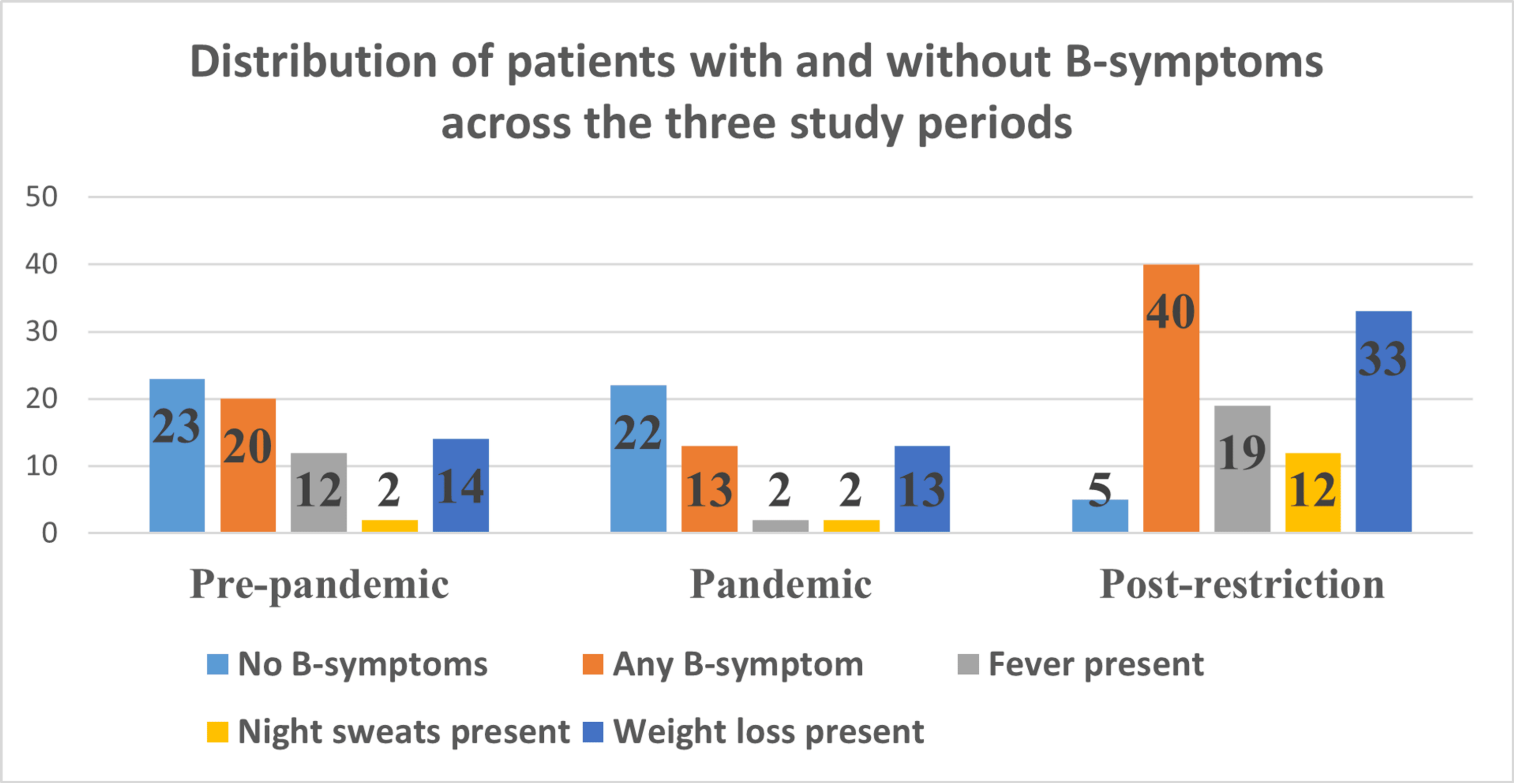
Distribution of patients with and without B-symptoms across the three study periods Percentages were calculated within each temporal subgroup. Created using Microsoft Excel 2016 for Windows

A clear shift toward more advanced disease at presentation was observed across the three study periods (Table 2), with early-stage disease being proportionally less frequent after the lifting of restrictions and advanced stages becoming more prominent in the post-restriction interval. Stage I disease was recorded only in the pre-pandemic group, early-stage disease overall decreased from 27.9% and 31.4% in the first two periods to 11.1% after restrictions, and stage IV increased from 37.2% and 37.1% before and during the pandemic to 48.9% thereafter. A similar redistribution was seen in the symptom profile; the proportion of patients without B-symptoms markedly decreased to 11.1% in the post-restriction group, while fever, night sweats, weight loss, and more complex symptom constellations became more frequent (Figure 3).

**Table 2:** Stage at diagnosis and B-symptom burden across the three study periods Data are presented as numbers (%) within each study period. Ann Arbor substages were grouped into major stage categories to improve clarity and comparability. B-symptoms were defined as fever > 38°C, drenching night sweats, and unintentional weight loss > 10% within the preceding six months. Symptom-specific rows indicate the presence of each symptom alone or in combination with other B-symptoms.

Clinical presentation at diagnosis	Pre-pandemic (n = 43)	Pandemic (n = 35)	Post-restriction (n = 45)
Stage I	1 (2.3%)	0 (0.0%)	0 (0.0%)
Stage II	11 (25.6%)	11 (31.4%)	5 (11.1%)
Stage III	9 (20.9%)	8 (22.9%)	12 (26.7%)
Stage IV	16 (37.2%)	13 (37.1%)	22 (48.9%)
Unspecified stage	6 (14.0%)	3 (8.6%)	6 (13.3%)
Early stage I to II	12 (27.9%)	11 (31.4%)	5 (11.1%)
Advanced stage III to IV	25 (58.1%)	21 (60.0%)	34 (75.6%)
No B-symptoms	23 (53.5%)	22 (62.9%)	5 (11.1%)
Any B-symptom	20 (46.5%)	13 (37.1%)	40 (88.9%)
Fever present	12 (27.9%)	2 (5.7%)	19 (42.2%)
Night sweats present	2 (4.7%)	2 (5.7%)	12 (26.7%)
Weight loss present	14 (32.6%)	13 (37.1%)	33 (73.3%)

Baseline demographic and social characteristics

The demographic profile remained broadly comparable across the three study periods (Table 3). No statistically significant differences were observed for sex distribution, area of residence, or occupational status, although several descriptive trends deserve note. Patients diagnosed in the post-restriction interval tended to be older, more frequently urban, and more often represented by socially vulnerable categories; the proportions of patients with no formal education and of those no longer married increased descriptively across time.

**Table 3:** Baseline demographic and selected social characteristics of the study population Data are presented as numbers (%) unless otherwise stated. P values are shown only for variables that underwent formal between-group comparison. Categories retained purely for descriptive completeness were not formally tested because of missingness or sparse data. SD: standard deviation

Variable	Pre-pandemic (n = 43)	Pandemic (n = 35)	Post-restriction (n = 45)	P value
Male sex	25 (58.1%)	16 (45.7%)	21 (46.7%)	0.452
Female sex	18 (41.9%)	19 (54.3%)	24 (53.5%)	
Age, mean ± SD, years	59.74 ± 13.86	61.69 ± 15.90	66.33 ± 13.07	0.08
Urban residence	23 (53.5%)	21 (60.0%)	32 (71.1%)	0.23
Rural residence	20 (46.5%)	14 (40.0%)	13 (28.9%)	
Employed	13 (30.2%)	10 (28.6%)	9 (20.0%)	0.55
Retired	25 (58.1%)	17 (48.6%)	27 (60.0%)	
Unemployed	5 (11.6%)	8 (22.9%)	9 (20.0%)	
No formal education	0 (0.0%)	6 (17.1%)	13 (28.9%)	-
Married	23 (53.5%)	5 (14.3%)	8 (17.8%)	-

Associated comorbidities

Comorbidities were frequent across all three periods. Cardiovascular disease represented the dominant category overall, and hypertension remained the most common individual comorbidity in each interval (Table 4). At the same time, selected pulmonary, cerebrovascular, hepatic, metabolic, and musculoskeletal conditions appeared more often in the post-restriction group, suggesting that, by the time patients re-entered hematology care after the most disruptive phases of the pandemic, they may also have been carrying a somewhat heavier burden of concomitant chronic disease.

**Table 4:** Selected associated comorbidities across the three study periods Data are presented as numbers (%) within each study period and overall. Percentages in the Overall column use the full cohort (N = 123) as the denominator. Only the most recurrent or clinically relevant comorbidities were retained in the main table. Comorbidities were not mutually exclusive, and individual patients could contribute to more than one category

Comorbidity	Pre-pandemic (n = 43)	Pandemic (n = 35)	Post-restriction (n = 45)	Overall (N = 123)
Hypertension	16 (37.2%)	16 (45.7%)	19 (42.2%)	51 (41.5%)
Ischemic heart disease	7 (16.3%)	7 (20.0%)	1 (2.2%)	15 (12.2%)
Pulmonary infections	1 (2.3%)	9 (25.7%)	4 (8.9%)	14 (11.4%)
Chronic pulmonary disease	5 (11.6%)	5 (14.3%)	5 (11.1%)	15 (12.2%)
Liver disease	6 (14.0%)	6 (17.1%)	10 (22.2%)	22 (17.9%)
Gastroduodenal disease	8 (18.6%)	8 (22.9%)	9 (20.0%)	25 (20.3%)
Cerebrovascular disease	3 (7.0%)	3 (8.6%)	10 (22.2%)	16 (13.0%)
Diabetes mellitus	8 (18.6%)	8 (22.9%)	12 (26.7%)	28 (22.8%)
Osteoarthritis and spondylosis	7 (16.3%)	7 (20.0%)	9 (20.0%)	23 (18.7%)
Vertebral collapse or fractures	0 (0.0%)	2 (5.7%)	3 (6.7%)	5 (4.1%)

Laboratory findings

Baseline laboratory differences across periods were limited (Table 5). Hemoglobin and red cell parameters showed only a mild downward trend from the pre-pandemic to the post-restriction interval, while platelet counts remained broadly comparable. By contrast, leukocyte counts tended to be higher after restrictions, and lactate dehydrogenase showed greater variability during the pandemic interval, findings that may be directionally compatible with more heterogeneous clinical presentation, although not sufficiently specific to define an autonomous biological signature of delayed diagnosis.

**Table 5:** Comparisons of selected laboratory findings at diagnosis and at the last available evaluation across the three study periods Values are expressed as mean ± SD. P values refer to cross-period comparisons at each evaluation time point. The number of available observations varied by parameter and study period because not all patients had complete laboratory data at both time points. LDH: lactate dehydrogenase, SD: standard deviation *Statistically significant (P < 0.05)

Parameter	At diagnosis, pre-pandemic	At diagnosis, pandemic	At diagnosis, post-restriction	P value	Last available, pre-pandemic	Last available, pandemic	Last available, post-restriction	P value
Hemoglobin (g/dL)	12.76 ± 1.97	12.28 ± 1.72	12.16 ± 2.14	0.34	11.59 ± 1.55	11.89 ± 1.66	11.47 ± 1.88	0.57
White blood cell count (×10³/μL)	9.17 ± 5.78	8.52 ± 4.26	14.00 ± 13.40	0.19	7.20 ± 6.12	9.68 ± 11.56	9.25 ± 8.36	0.23
Platelet count (×10³/μL)	279.70 ± 151.14	248.53 ± 123.59	245.60 ± 122.93	0.85	253.83 ± 140.73	255.13 ± 108.45	254.36 ± 199.41	0.48
LDH (U/L)	255.22 ± 153.92	381.25 ± 466.97	253.53 ± 117.32	0.86	261.95 ± 91.28	262.03 ± 152.42	255.10 ± 135.45	0.30
Total bilirubin (mg/dL)	0.66 ± 0.35	0.65 ± 0.26	0.87 ± 0.59	0.30	0.64 ± 0.59	0.62 ± 0.37	1.82 ± 4.26	0.003*
Creatinine (mg/dL)	0.84 ± 0.17	1.16 ± 1.08	0.91 ± 0.28	0.16	0.77 ± 0.16	1.21 ± 1.20	1.12 ± 0.50	0.002*
Total cholesterol (mg/dL)	161.14 ± 70.65	192.95 ± 64.13	176.17 ± 50.12	0.33	182.56 ± 27.55	236.80 ± 102.75	294.47 ± 140.64	0.01*
Triglycerides (mg/dL)	171.33 ± 179.72	202.00 ± 151.78	113.83 ± 72.96	0.03	171.42 ± 72.67	209.00 ± 98.35	374.04 ± 190.05	<0.0001*

Paired analysis between diagnosis and the last available evaluation showed several within-period changes (Table 6). Hemoglobin and hematocrit declined significantly in the pre-pandemic and post-restriction groups, while leukocyte counts decreased significantly in the same intervals, suggesting partial correction of baseline abnormalities over time. By contrast, platelet count and lactate dehydrogenase did not show significant paired shifts, whereas creatinine, cholesterol, and triglycerides displayed period-specific changes that are likely to reflect not only disease burden but also treatment exposure, comorbidity, and follow-up structure.

**Table 6:** Paired evolution of selected laboratory parameters between diagnosis and the last available evaluation within each study period Values are expressed as mean ± SD. P values refer to paired within-period comparisons between diagnosis and the last available evaluation. The number of paired observations varied by parameter and by study period. NA: not applicable because a formal comparison was not retained in the source dataset due to the very small number of available paired values, LDH: lactate dehydrogenase, SD: standard deviation *Statistically significant (P < 0.05)

Parameter	Diagnosis, pre-pandemic	Last available, pre-pandemic	P value	Diagnosis, pandemic	Last available, pandemic	P value	Diagnosis, post-restriction	Last available, post-restriction	P value
Hemoglobin (g/dL)	12.76 ± 1.97	11.59 ± 1.55	0.0005	12.21 ± 1.79	11.88 ± 1.69	0.26	12.16 ± 2.14	11.54 ± 1.89	0.026*
White blood cell count (×10³/μL)	9.17 ± 5.78	7.20 ± 6.12	0.01	8.54 ± 4.49	9.77 ± 11.74	0.52	14.73 ± 13.47	9.89 ± 8.72	0.01*
Platelet count (×10³/μL)	281.71 ± 152.38	253.83 ± 140.73	0.50	253.17 ± 129.78	255.28 ± 110.26	0.89	241.20 ± 121.06	250.90 ± 195.10	0.74
LDH (U/L)	253.73 ± 151.57	268.15 ± 94.78	0.27	404.67 ± 501.02	255.41 ± 156.91	0.29	258.44 ± 119.96	253.56 ± 134.49	0.46
Creatinine (mg/dL)	0.84 ± 0.15	0.76 ± 0.16	0.0008	1.21 ± 1.21	1.21 ± 1.20	0.80	0.88 ± 0.24	1.11 ± 0.51	0.01*
Total cholesterol (mg/dL)	165.44 ± 62.46	188.11 ± 31.99	0.37	270.50 ± 19.82	243.75 ± 117.28	NA	174.12 ± 46.58	294.00 ± 148.25	<0.0001*
Triglycerides (mg/dL)	128.92 ± 79.33	161.75 ± 69.82	0.23	148.20 ± 76.28	190.40 ± 90.86	0.25	115.00 ± 96.23	345.67 ± 225.71	0.008*

Treatment patterns

Treatment patterns remained centered on standard chemoimmunotherapy across all three study periods (Table 7). Rituximab, cyclophosphamide, doxorubicin, vincristine, and prednisone (R-CHOP) was the most frequently used regimen in every interval, and rituximab, cyclophosphamide, idarubicin, vincristine, and prednisone (R-CZOP) occupied the secondary position. Although the post-restriction period showed a somewhat broader therapeutic spectrum, with the appearance of methotrexate, cytarabine, thiotepa, and rituximab (MATRIX) and polatuzumab-based combinations, the therapeutic backbone of care remained broadly stable over time.

**Table 7:** Recorded treatment regimens across the three study periods Data are presented as numbers (%) relative to the number of patients in each study period and overall. Percentages in the Overall column use the full cohort (N = 123) as the denominator. Treatment categories were not necessarily mutually exclusive because some patients received more than one regimen during follow-up. This table is presented descriptively; no formal hypothesis testing was performed because of overlapping exposure categories and small counts in several regimens. R-CHOP: rituximab, cyclophosphamide, doxorubicin, vincristine, and prednisone, R-CZOP: rituximab, cyclophosphamide, idarubicin, vincristine, and prednisone, CZOP: cyclophosphamide, idarubicin, vincristine, and prednisone, R-DHAP ± G: rituximab, dexamethasone, high-dose cytarabine, and cisplatin with or without granulocyte colony-stimulating factor, R-COP: rituximab, cyclophosphamide, vincristine, and prednisone, EPOCH ± R: etoposide, prednisone, vincristine, cyclophosphamide, and doxorubicin with or without rituximab, MATRIX: methotrexate, cytarabine, thiotepa, and rituximab

Treatment regimen	Pre-pandemic (n = 43)	Pandemic (n = 35)	Post-restriction (n = 45)	Overall (N = 123)
R-CHOP	20 (46.5%)	17 (48.6%)	23 (51.1%)	60 (48.8%)
R-CZOP	7 (16.3%)	8 (22.9%)	8 (17.8%)	23 (18.7%)
CZOP	2 (4.7%)	2 (5.7%)	3 (6.7%)	7 (5.7%)
R-DHAP ± G	4 (9.3%)	3 (8.6%)	2 (4.4%)	9 (7.3%)
R-COP	4 (9.3%)	4 (11.4%)	2 (4.4%)	10 (8.1%)
EPOCH ± R	3 (7.0%)	1 (2.9%)	1 (2.2%)	5 (4.1%)
Hyper CVAD	2 (4.7%)	2 (5.7%)	1 (2.2%)	5 (4.1%)
MATRIX	0 (0.0%)	0 (0.0%)	3 (6.7%)	3 (2.4%)
Polatuzumab bendamustine ± R	0 (0.0%)	0 (0.0%)	2 (4.4%)	2 (1.6%)
Other rare regimens	6 (14.0%)	6 (17.1%)	4 (8.9%)	16 (13.0%)

## Discussion

This study suggests that the effect of the COVID-19 period on newly diagnosed B-NHL was not limited to a temporary fluctuation in case numbers but extended to the clinical profile of the patients who ultimately reached specialist care, because, although the number of newly diagnosed cases numerically recovered after the pandemic interval, the post-restriction group in our cohort presented with a clear shift toward more advanced disease and a markedly heavier B-symptom burden, recovery proving therefore to be quantitative rather than fully clinical and fitting the broader post-pandemic concern, raised across oncologic settings [6-12], that nominal service normalization may still conceal delayed and clinically less favorable case capture [13-16].

That interpretation is consistent with the broader oncology literature showing that the first pandemic waves were accompanied by fewer cancer diagnoses [8,9], disrupted diagnostic pathways [7,10,12], and concern for later stage effects, modeling work from England predicting avoidable excess cancer deaths attributable to diagnostic delay [6], while population-based analyses from Belgium [7], the United States [9,10], Alberta [11], Portugal [16], and European registry networks [12] documented substantial drops in diagnoses, incomplete recovery in some settings, and shifts in stage distribution or short-term outcomes. A recent meta-analysis estimating an overall 23% decline in cancer diagnoses during the pandemic [8] further reinforces the plausibility that backlog effects could re-emerge later as more clinically overt disease, and a major systematic review showing increased mortality with each four-week treatment delay across several oncologic settings [15] underscores the general clinical relevance of timeliness.

Our lymphoma-specific findings likewise align with the limited but relevant comparative literature. The Swedish Lymphoma Register showed that fewer patients were diagnosed during March-June 2020 and that a larger proportion presented with stage IV disease in 2021 [13], while Küçükyurt et al. reported, in classical Hodgkin lymphoma, a doubling of the median symptom-to-diagnosis interval during the pandemic from 8 to 16 weeks, with a directionally higher proportion of advanced-stage disease [14]. Beyond the pandemic context itself, however, lymphoma diagnostic pathways are intrinsically complex; qualitative and population-based studies demonstrated that symptom appraisal [17], emergency presentation routes and diagnostic inequalities [18], structural barriers in diffuse large B-cell lymphoma [19], and background comorbidity burden [20,21] may all prolong the route to diagnosis in aggressive and indolent lymphomas alike; although our cohort addresses B-NHL rather than Hodgkin lymphoma and does not directly quantify delay in days or weeks, the observed post-restriction stage migration remains compatible with the same mechanism of deferred evaluation and delayed specialist capture [22,23].

The B-symptom profile in our cohort strengthens that interpretation: patients without systemic symptoms remained common before and during the pandemic but became distinctly uncommon after restrictions were lifted, whereas weight loss, fever, night sweats, and more complex symptom constellations became substantially more frequent in the post-restriction interval. Because the subtype spectrum remained broadly stable across the three periods, this pattern is difficult to explain by histopathologic redistribution alone and instead suggests that a proportion of patients re-entered the diagnostic pathway later, after disease had become more clinically manifest, a scenario that is biologically and clinically plausible in lymphoma, where the initially nonspecific nature of symptoms [17], the possibility of non-specialist entry routes [18], and the coexistence of competing comorbid explanations [20,21] may prolong both help-seeking and diagnostic intervals [22,23].

The laboratory findings were more nuanced than the staging results and should therefore be interpreted cautiously, because most hematologic and biochemical variables did not show a uniform cross-period signature of delayed presentation, even though post-restriction patients tended to have higher baseline leukocyte counts and the pandemic interval showed greater lactate dehydrogenase variability. These observations may be directionally compatible with more heterogeneous or more advanced disease, yet they are not sufficiently specific to serve as independent proof of diagnostic delay and are better understood as supportive secondary findings embedded within a broader clinicopathologic pattern.

The comorbidity profile provides additional context. Cardiovascular and metabolic disease dominated across all three periods, which is unsurprising given the age structure of the cohort, but selected cerebrovascular, hepatic, diabetic, and musculoskeletal conditions were more frequently recorded after restrictions, a pattern that may indicate that patients diagnosed later in the study period arrived at hematology care with greater overall clinical fragility. Such background vulnerability matters in lymphoma care, especially during systemic disruption, because it can complicate workup, intensify symptom burden, and narrow therapeutic margins [24-26]; moreover, population-level analyses have shown that multimorbidity is highly prevalent at the time of cancer diagnosis [20] and may shape both diagnostic routes and treatment delivery [18,21], reinforcing the plausibility that healthcare disruption and comorbidity burden interacted rather than acting in isolation in our cohort.

Treatment patterns in our cohort remained anchored in standard chemoimmunotherapy, with R-CHOP being the dominant regimen throughout, which is clinically relevant because international lymphoma guidance issued during the pandemic emphasized preserving curative-intent therapy whenever feasible and avoiding unnecessary deferral of essential treatment [27]. At the same time, observational studies of lymphoma and COVID-19 have shown that active lymphoma itself, rather than recent treatment alone, is a major determinant of adverse outcomes [28,29], a conclusion reinforced by broader reviews of lymphoid malignancies in the SARS-CoV-2 era [24-26] and one that supports the importance of timely diagnosis and disease control. Additional lymphoma-specific data suggest that the diagnosis-to-treatment interval may also carry prognostic information in diffuse large B-cell lymphoma [23,30], although its interpretation remains complex because the shortest intervals frequently characterize the sickest patients presenting with the highest-risk disease [19,22]. The relative continuity of first-line treatment structure in our center is therefore reassuring, yet it could not fully compensate for later presentation at diagnosis.

Strengths and limitations

This study has several limitations. Its retrospective design makes it vulnerable to incomplete documentation, missing values, and heterogeneity in record quality, while its single-center setting limits generalizability and the sample size restricts the precision of subgroup analyses, particularly for rare histopathologic entities and laboratory comparisons with limited paired data. The requirement for at least two standard inpatient admissions may also have preferentially retained more complex cases; in addition, we did not have consistent patient-level data on exact referral delay, biopsy waiting time, prior SARS-CoV-2 infection, vaccination status, cause-specific mortality, or care delivered outside the hospital system, and the relationship between pandemic disruption and stage shift should therefore be interpreted as compatible and plausible rather than directly proven.

The study nevertheless has relevant strengths, evaluating a clearly defined cohort of newly diagnosed patients with B-NHL across three clinically meaningful temporal intervals within the same institutional environment and examining not only case numbers but also stage, symptom burden, comorbidities, laboratory parameters, and treatment patterns. By combining these dimensions within a single institutional dataset, the study renders the post-pandemic signal clinically interpretable rather than merely descriptive and offers a useful local perspective from a Romanian tertiary center, one that complements broader registry-based literature while also suggesting that future multicenter work should integrate patient-pathway metrics, referral intervals, biopsy waiting times, and comorbidity-adjusted analyses so that the mechanisms underlying stage migration may be reconstructed more precisely.

## Conclusions

In this retrospective single-center study, the COVID-19 period was followed not only by a temporary reduction in newly diagnosed B-cell non-Hodgkin lymphoma cases but also by a subsequent shift toward more advanced and more symptomatic disease at presentation, numerical recovery after the lifting of major restrictions not being matched by comparable clinical normalization; the relative stability of the histopathologic spectrum further argues that the dominant effect was less a redistribution of subtype biology than a later point of entry into the diagnostic pathway, with patients reappearing in care after disease had become more overt clinically. Taken together, these findings support the need for resilient hematology pathways capable of preserving timely lymphoma diagnosis even during periods of major health system stress, while also indicating several concrete directions for future research, including multicenter validation in larger Romanian or regional cohorts, incorporation of referral and biopsy waiting time metrics, integration of vaccination and prior infection data, linkage with survival and relapse outcomes, and development of pathway-level interventions designed to shorten the interval from first symptom recognition to specialist hematology assessment, thereby clarifying which component of delay most strongly drives post-disruption stage migration.
